# Within What Distance Does “Greenness” Best Predict Physical Health? A Systematic Review of Articles with GIS Buffer Analyses across the Lifespan

**DOI:** 10.3390/ijerph14070675

**Published:** 2017-06-23

**Authors:** Matthew Browning, Kangjae Lee

**Affiliations:** 1 Department of Recreation, Sport and Tourism, University of Illinois at Urbana-Champaign, Champaign, IL 61820, USA; 2 Illinois Informatics Institute, University of Illinois at Urbana-Champaign, Champaign, IL 61802, USA; klee171@illinois.edu

**Keywords:** systematic review, greenness, Geographic Information System (GIS), physical health, buffers, green space, park, health outcomes, Normalized Difference Vegetation Index (NDVI)

## Abstract

Is the amount of “greenness” within a 250-m, 500-m, 1000-m or a 2000-m buffer surrounding a person’s home a good predictor of their physical health? The evidence is inconclusive. We reviewed Web of Science articles that used geographic information system buffer analyses to identify trends between physical health, greenness, and distance within which greenness is measured. Our inclusion criteria were: (1) use of buffers to estimate residential greenness; (2) statistical analyses that calculated significance of the greenness-physical health relationship; and (3) peer-reviewed articles published in English between 2007 and 2017. To capture multiple findings from a single article, we selected our unit of inquiry as the analysis, not the article. Our final sample included 260 analyses in 47 articles. All aspects of the review were in accordance with PRISMA guidelines. Analyses were independently judged as more, less, or least likely to be biased based on the inclusion of objective health measures and income/education controls. We found evidence that larger buffer sizes, up to 2000 m, better predicted physical health than smaller ones. We recommend that future analyses use nested rather than overlapping buffers to evaluate to what extent greenness *not* immediately around a person’s home (i.e., within 1000–2000 m) predicts physical health.

## 1. Introduction

As a result of rapid urbanization, a growing disconnect from nature, and rising rates of disease and illness, many groups are increasingly interested in the effects of greenspaces on physical health. Greenspaces provide daily opportunities for physical exercise and stress relief [1]. The distance within which greenspaces provide these opportunities to any meaningful degree, however, is not well understood. Is there a stronger correlation between health and greenspaces within 250 m of someone’s home, than those within 1000 m of someone’s home? The empirical evidence needed to answer such questions is lacking.

Our current understanding of the distance within which greenness matters is based largely on expert opinion. In the United States, some practitioners suggest that having greenspace within a five-minute walk (approximately 0.2 miles or 0.32 km) is most important for physical health, while others assert that a 0.50- or 0.75-mile (0.80 or 1.20 km) range is more accurate [2]. A better understanding of the maximum distance to consider when analyzing greenspace would help urban planners and public health officials better evaluate how to design minimum standards for greenspaces near members of their communities.

Geographic information system (GIS) software packages (i.e., ArcGIS, ESRI Inc., Redlands, CA, USA) provide powerful tools to provide empirical evidence on how different distances in which greenness is measured impact physical health. A multitude of remote sensing and GIS datasets (i.e., Normalized Difference Vegetation Index (NDVI), land cover datasets, and park layers) can be uploaded into these packages to provide objective measures of green cover in countries around the world [1]. Buffer tools (the “Buffer (Analysis)” toolkit in ArcGIS) can then calculate the percentage of greenspace—or relative “greenness”—within a specified geographic polygon, for example, the area surrounding a person’s house.

The results of at least three studies using GIS buffer tools provide some evidence that greenness at *any* buffer size predicts physical health equally well. A national survey of physical activity and urban greenspace in Canada, for example, found that 30 m buffers predicted health as well as 500 m buffers [3]. Another study of greenness and air pollution exposure during pregnancy found that 100 m, 250 m, and 500 m buffers predicted birth and development outcomes similarly well [4]. A second study on birth and development outcomes and greenness found that 100 m and 500 m buffers had similar results [5]. What these studies fail to answer, however, is, does the impact of greenness on physical health plateau at a particular buffer size? To answer this question, one can compile these findings with those of other studies that examine greenness within larger buffer zones and look for trends across the studies.

A review of GIS buffer studies is valuable despite the recent advances in activity tracking that provide potentially more accurate measurements of the natural/built environment available to people [6]. For example, tracking with global positioning system (GPS) units can accompany individuals’ whose physical activity is being tracked. The trajectories recorded from these units help to create neighborhood areas that encompass trips taken by individuals throughout their everyday life. As such, the area in which greenness is examined with GPS units includes both the residential and non-residential environments to which individuals are exposed—such as farther-away but regularly-visited parklands—and therefore considers the critical factor pertaining to which greenspaces are accessible along transportation routes [7]. Buffers created with distances set by researchers in GIS systems, in contrast, are artificially created and may over or under-estimate the range available to—and used by—residents. On the other hand, GPS is an approach being adopted in an only limited number of public health fields of research, primarily physical activity research (i.e., [8,9,10,11]), and several recent studies still use artificially-created buffers (i.e., [12,13,14]). Providing every individual in a sample population with GPS units is not feasible for all study designs, geographic contexts, study populations, and sample sizes. In order to review the available evidence on greenness around a person’s home, it is, therefore, important to conduct a systematic review of artificially-created buffers in GIS.

We reviewed the body of literature that uses greenness buffer analyses to better understand at what distance greenness best predicts physical health. To identify this body of work, we included only those analyses that (1) used GIS buffers to estimate residential greenness; (2) used statistical analyses that calculated the significance of the greenness-physical health relationship; and (3) were peer-reviewed articles published in English between 2007 and May 2017. Our objective was to identify which buffer sizes were supported by the literature as having the strongest theoretically expected relationships between greenness and physical health—that is, findings where greater greenness correlated with better physical health.

## 2. Methods

All steps in our review process were conducted in accordance with the Preferred Reporting Items for Systematic Reviews and Meta-Analyses (PRISMA) guidelines [15]. The screening and analyzing of data were conducted in Microsoft Excel for Mac.

### 2.1. Literature Search

We searched Web of Science to identify relevant articles published in English between 2007 and May 2017. Search terms included “greenness” measures, GIS datasets and analytical techniques, and physical health outcomes (see Appendix A, Table A1 for a list of all terms).

### 2.2. Selection Criteria

To extract data from multiple analyses included in articles, we conducted a two-step selection process. First, we selected those articles with an overall focus and at least one reported analysis that was relevant for this review. Next, we selected the relevant analyses from each of these articles.

#### 2.2.1. Article Selection

Initial eligibility of articles was determined by reviewing their titles and abstracts. The full text of the remaining articles was then screened for three criteria. First, the focus of the article had to measure the impact of greenness on physical health using GIS buffer analyses. “Greenness” could include percent green cover, green space, or parks; the number of green spaces, parks, or open space within the buffer; or the standard deviation of greenness or landscape indices. Second, the authors conducted inferential statistical analyses to measure the relationship between these variables; and, third, statistical significance tests (*p*-values) resulting from these analyses were performed.

#### 2.2.2. Analysis Selection

Next, we screened analyses within eligible articles to determine which analyses were appropriate to examine in this review. Screening criteria included: (1) GIS buffers used residential center points in GIS buffer analyses, rather than school or workplace center points; and (2) the dependent variable was a physical, not a mental health outcome. No exclusionary criteria were set for the range of ages studied. Analyses included, therefore, used buffers around physical addresses of homes or centers of politically defined geographic areas enclosing homes (i.e., census tracts) and surveyed sample ages across the lifespan, from infants to elders.

### 2.3. Data Extraction

Most data were extracted at the analysis, not the article, level. Only first author, publication year, and article title were identified at the article level. In contrast, a wide range of characteristics were identified at the analysis level, including article information (first author, publication year, and article title); greenness type (“greenness”, tree canopy, greenspace or open space, or park); physical health outcome; objectivity of physical health outcome; country/countries covered by sample population; age range(s) of sample population; size of sample; confounds included (i.e., age, sex, race, income, employment, education, urbanity, etc.); presence of spatial autocorrelation test(s); center point of GIS buffer (home, postal code, or census tracts/blocks); type of GIS buffer (see below); size of buffer in meters; presence or absence of statistically significant association between health outcome and greenness; and direction of significant associations between health outcome and greenness.

**Figure 1:**
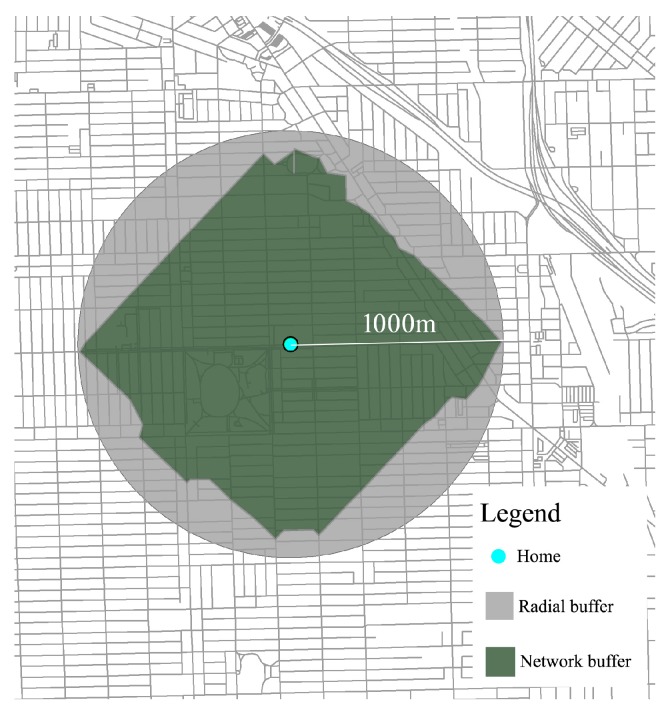
Buffer analyses are tools to calculate the “greenness” of residential environments. Buffers are drawn two ways. *Radial buffers* (outer circle in gray) show greenness in the circle of a specified radius around a center point—in this case, 1000 m. *Network buffers* (inner polygon shape in green) show greenness in the region within a specified walking or driving distance of a center point.

The type of buffer data extracted concerned whether the buffer was made in one of two ways. *Radial buffers* estimate greenness “as the crow flies” around a point. In this case, when the researcher specified a radial distance, the GIS software drew a circle (circular buffer) with a radius equal to that value. The circle was centered on a specified point—often an individual’s home address or the center of a geographic zone within which the individual lives (i.e., census tract or postal code). In contrast, *network buffers* estimate greenness along transportation pathways. The GIS software determines the network buffers, after the researcher specifies the distance, along all available walking or driving routes, based on road network GIS data. This process results in a polygon (network buffer), which is not perfectly circular in shape, because the perimeter of the buffer represents points that are within a set distance along trails and roads (Figure 1).

Several analytical characteristics were reported in detail in articles, and we coded these into broader categories for this review. Physical health outcomes were divided into 18 categories. These were adapted from categories identified in previous greenness and health literature reviews [1,16,17]. The manner of data collection for physical health outcomes was grouped into three categories, based on whether data were *objective* (i.e., biomarkers, vital signs), *expert or clinician diagnoses* (i.e., electronic medical records), or *subjective* (i.e., self-reported health questionnaires). Lastly, we grouped age ranges into five categories: samples with individuals less than one year old (*infants*), 2–10 years old (*children)*, 11–17 years old (*youth)*, 18–79 (*adults)*, and 80 years and over (*elders)*.

### 2.4. Evaluation of Possible Bias

Our focus was on evaluating analyses with higher or lower levels of potential bias. The items we evaluated were individual *analyses,* not *articles.* Thus, our evaluative judgments in this review do not cover the articles in their entirety. It is possible that any given article had some analyses that could be judged as having higher potential bias and others as lower.

To identify possible criteria that would demonstrate potential bias, we consulted criteria used in prior systematic literature reviews on greenness and human health outcomes [18,19]. We found that some prior criteria (i.e., multiplicity of outcome variables within a single article) did not apply to our unit of examination—that is, analyses rather than articles—and other criteria represented characteristics of potential bias which we had already excluded in our screening process (i.e., expert assessments of “greenness” versus land-cover maps or satellite system assessments). We narrowed our criteria to two items: did the analysis include a subjective measure of physical health as its dependent variable; and did the analysis account for *the* critical confounding variable—income? We chose the latter criteria because a robust body of work demonstrates that this variable, more than any other, partially explains the relationship between greenness and health outcomes [1,16]. Not including a measure of income—such as socio-economic status or the common proxy, level of education—overestimates positive relationships between greenness and health.

Our chosen criteria for assessing bias (subjective measures of health and controls for socio-economic status) allowed us to rank articles into three levels. We designated analyses that did not meet *either* of the criteria as *more likely to be biased.* We designated analyses that met *one* criteria as *less likely to be biased* and those that met *both* criteria as *least likely to be biased.*

## 3. Results

### 3.1. Subsection

**Figure 2:**
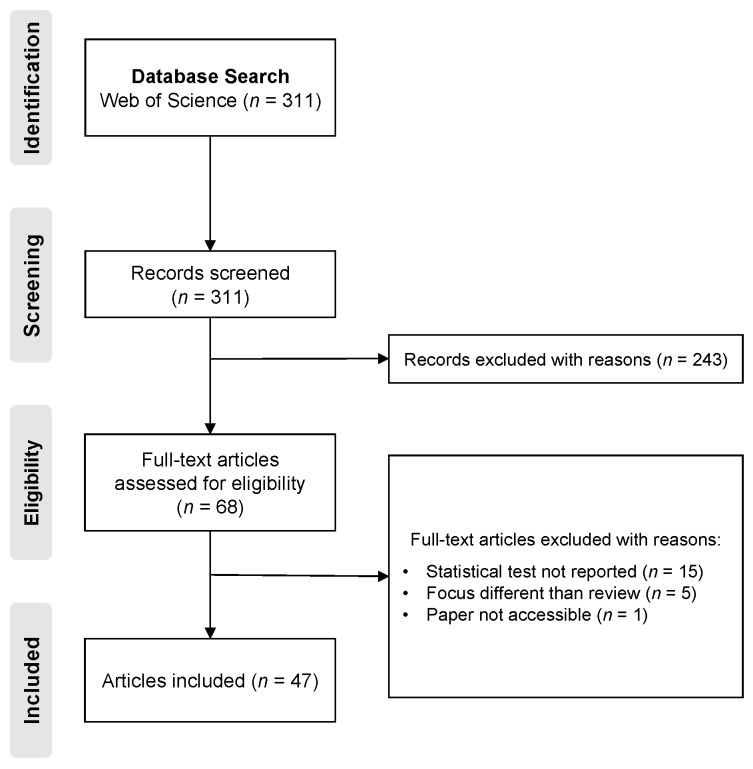
Flow diagram of the screening of articles considered for inclusion in this review - conducted using the PRISMA process.

The initial database search produced 311 records, but the majority were not salient for this review. The full texts of 68 articles were reviewed for eligibility, and 47 met our criteria for inclusion (see Figure 2 for details on articles removed at each step of the screening process).

Articles contained between one [20] and 44 [21] analyses that met our inclusion criteria. In total, 260 analyses were included in this review (for a complete listing of articles and their analyses, see Appendix A, Table A2).

### 3.2. Descriptive Characteristics of Articles

**Figure 3:**
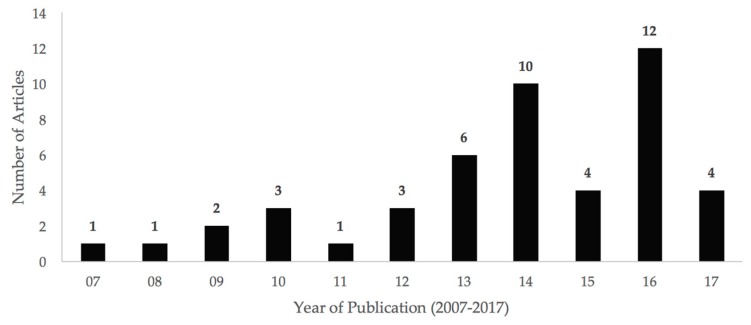
The number of articles using GIS buffer analyses to estimate the impact of greenness on physical health has increased over time, especially since 2013. Data for 2017 is incomplete because the review only included articles published before May 2017.

We found that the number of published articles on the use of GIS buffers to estimate the impact of greenness on physical health increased over time. The majority of articles identified in this review were published in the last four years (Figure 3).

**Figure 4:**
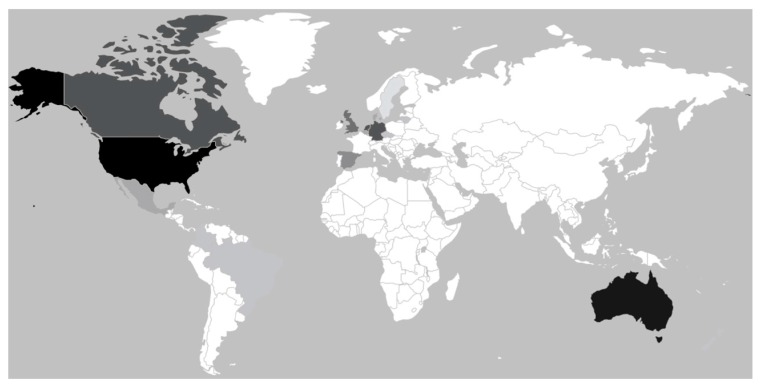
Articles studied populations from 17 countries, with the United States and Australia most commonly studied. Darker colors represent more articles on populations from that country.

We also found that articles examined populations from around the world. In total, 17 countries were represented: Australia, Belgium, Brazil, Canada, Colombia, Czech Republic, Denmark, Germany, Hong Kong, Lithuania, Mexico, Netherlands, New Zealand, Spain, Sweden, the United Kingdom, and the United States (Figure 4). Populations from the United States and Australia were most frequently examined. The former was studied in nine articles and the latter in eight articles. The next most commonly studied populations were from the Netherlands, Germany, and Canada, which were examined in six articles each.

### 3.3. Descriptive Characteristics of Analyses

Analyses used a wide range of sample sizes and ages (Table 1). The smallest sample was 61 [22] and the largest was 345,143 [21]. Over two-thirds of analyses included at least 2500 individuals and one-third included at least 10,000. Adults were the most commonly studied age range, with 30% of analyses studying exclusively 18–79 year olds and another 36% studying 18–79 year olds as well as youth and/or elders.

Less than one-half of analyses (45%) displayed a likelihood of bias (Table 2), of which, less than 1% displayed a strong likelihood of bias. Nearly two-thirds (65%) included objective measures or expert/clinical diagnoses of physical health. Income measures were even more common: 85% of analyses included education as a proxy for income, and 17% included income measures.

Age and sex were additional common confounds, included in at least 80% of analyses. All other confounds were represented in less than one-third of analyses.

In regards to the methodological choices made in buffer analyses, we found that 73% of analyses used home addresses as buffer centers. Further, 17% of analyses controlled for spatial autocorrelation. A nearly equal number of analyses used “greenness” (48%) and/or green space or open space metrics (42%).

### 3.4. Consistency of Greenness-Physical Health Link

Only 35% of analyses (*n* = 91) demonstrated statistically significant relationships between greenness and improved physical health. The majority (62%, *n* = 161) demonstrated no significant relationship between these variables, and a few (3%, *n* = 8) even found a significant relationship in the opposite direction—more green being tied to worse physical health.

Findings were similar across the lifespan. Thirty-seven percent (*n* = 49) of the 131 analyses that studied only adult or adult and elder populations found significant positive effects of greenness, and 2% (*n* = 3) reported negative effects of greenness. Similarly, 29% (*n* = 22) of the 75 analyses that studied only children, youth, and/or infants—but not *only* infants—reported significant positive effects. Seven percent (*n* = 5) of these analyses reported negative effects.

### 3.5. Specific Physical Health Outcomes Studied

Three outcomes accounted for over one-half of the dependent variables studied in analyses. These commonly-studied outcomes included physical activity (the dependent variable in 25% of analyses, *n* = 65), birth and developmental outcomes (16%, *n* = 41), and cardiovascular outcomes (13%, *n* = 33). Obesity and atopy (asthma, allergies, and eczema) were also commonly studied; each was used in approximately 10% of analyses (*n* = 26 and 24, respectively). General health (i.e., self-reported health questionnaires asking respondents “in general, how would you rate your health?”) was studied in 6% (*n* = 16) of analyses. Twelve other physical health outcomes were studied in 5% or less of analyses each (*n* = 2 to 12).

Nine outcomes showed that greenness improved health in one-half or more of analyses, but most of these outcomes were studied just a few times (Table 3). Obesity and diabetes were the only two outcomes that improved with greenness in over one-half of a more substantial number of analyses (*n* > 10). Analyses using the three most commonly studied outcomes (physical activity, birth and developmental outcomes, and cardiovascular disease) found significant positive correlations between greenness and physical health just 18 to 34% of the time. When we examined the most commonly studied outcome (physical activity), we found that objective measurements using accelerometer devices showed significant associations almost twice as frequently as subjective, self-report measures.

### 3.6. Buffer Sizes Used

Buffers between 1000–1999 m in size were most commonly used. This size represented nearly one-third of all analysis (*n* = 84). The next most common were 500–999 m buffers, which were used in approximately one-quarter of analyses (*n* = 67). Other buffer size ranges (less than 250 m, 250 to 499 m, and greater than 2000 m) were used at similar frequencies: between 32 (12%) and 41 (16%) of analyses.

### 3.7. Buffer Size and Physical Health

**Figure 5:**
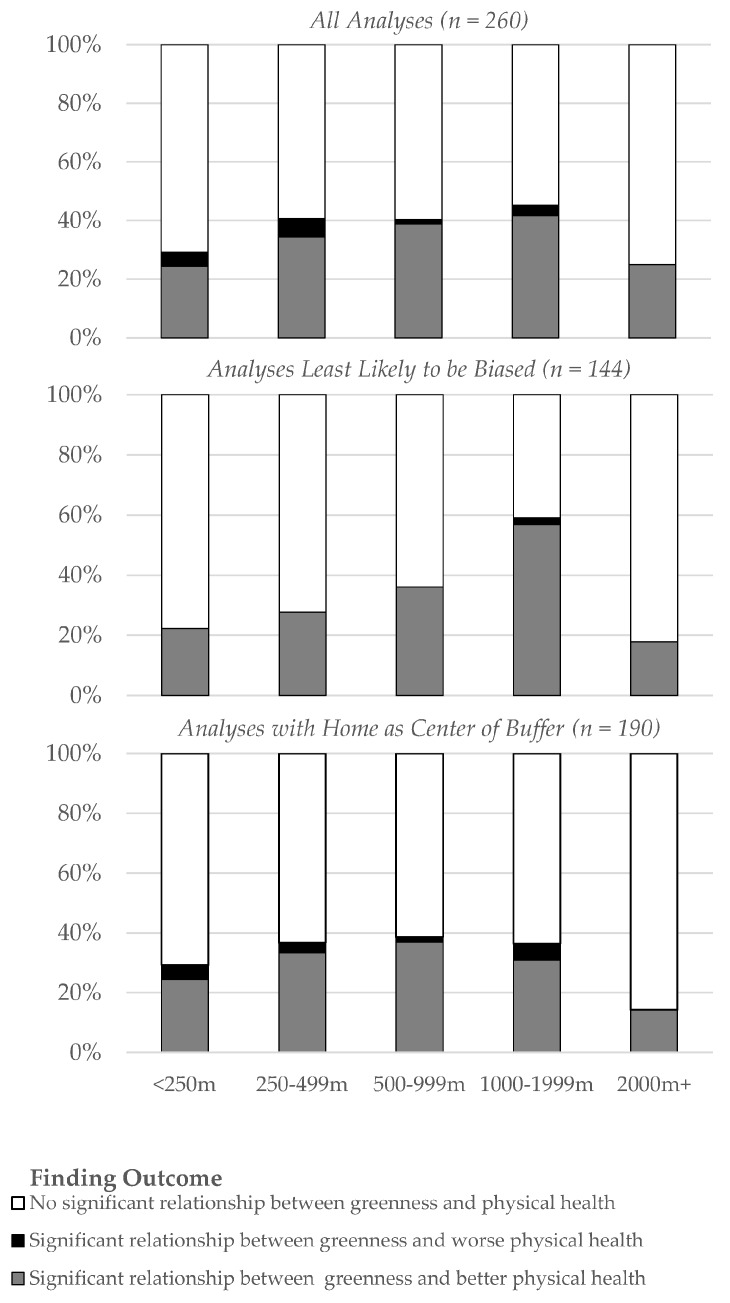
Percent of analyses showing statistically significant relationships between greenness and physical health improvement increases as buffer size increases, but only to a point. In all analyses reviewed (top), the percent of significant findings increased up to 1000–1999 m buffers, but then decreased at larger buffer sizes. This trend was exaggerated when examining only those analyses *least likely to be biased* (middle), as indicated by their use of objective health measures and the inclusion of income or education as a confounding factor. Analyses that used buffers centered on home addresses—rather than postal codes or census tracts—showed a different tipping point (bottom). In this subsample, analyses demonstrated that greenness improves physical health in buffers up to 500–999 m in size—not 1000–1999 m.

The relative number of analyses that tied greenness to physical health grew as the buffer size increased, but only up to 1999 m (Figure 5). In analyses with buffers of less than 250 m, 24% of studies reported that greenness improved physical health. This percent increased for analyses with buffers 250–499 m (34%), 500–599 m (39%) and 1000–1999 m (42%), but then dropped substantially for analyses with buffers of 2000 m or more (25%).

We tested whether this trend held with two subsamples of analyses, both of which included smaller but more reliable, valid measures of greenness. First, we restricted our sample to the 144 analyses that we judged as least likely to be biased. We found that the previously described trend was more pronounced in this subsample: the percent of analyses tying greenness to health increased from 22% with buffers of less than 250 m, to 57% for buffers between 1000–1999 m in size. Second, we restricted our sample to the 190 analyses that used individuals’ home addresses for buffer centers. This subsample again suggested that increasing numbers of analyses found positive ties between greenness and physical health as buffer size increases, but it plateaued at 500–999 m buffers. The percent of significant findings increased from 26% (buffers of less than 250 m) to 38% (buffers between 500–999 m), and then decreased to 33% (buffers between 1000–1999 m).

## 4. Discussion

The objective of this systematic literature review was to discover trends in buffer analyses reported in peer-reviewed journal articles on residential greenness and physical health. We identified 260 analyses in 47 articles that met our inclusion criteria.

These analyses showed the following trend: the likelihood of greenness predicting physical health increased as the size of the buffer increased. However, this trend plateaued at buffers between 1000–1999 m in size. Buffers of 2000 m and above were less likely to show greenness predicting physical health than all smaller buffers sizes.

This trend toward 1000–1999 m buffers best predicting physical health did *not* hold for the subsample of analyses that used home addresses as the center point of buffers. In 27% of the analyses in this review, researchers fixed buffers *not* around homes but around geographic midpoints of geographic regions (i.e., census tracts or postal codes). We examined trends when these analyses were excluded because buffers centered on homes were more reliable indicators of residential greenness than buffers centered on the center of the political boundary in which an individual resides. In this subsample, we found that increasing buffer sizes corresponded with increasing likelihood of greenness predicting physical health, and we further found that this trend plateaued at a certain size. The plateau size, however, was smaller than our earlier-found trend: buffers between 500–999 m in size and centered around homes best predicted physical health. Buffers smaller than 500 m and greater than 999 m were less likely to predict physical health.

These trends toward larger buffer size better predicting physical health do *not* indicate that nearby greenspace (i.e., a park located within a 250 m buffer from a house) is *less* predictive of physical health than distant greenspace. A 1000 m buffer around a house includes greenspace located in smaller, nested buffers around that house, as well as greenspace in distant edges of that buffer (i.e., a park 950 m away from buffer center). Thus, a 1000 m buffer includes all greenspaces captured in the 50 m-, 250 m-, and 500 m buffers. The extent of greenness for a 1000 m buffer, therefore, is the total green/open land uses within this circular area, divided by the total area of the buffer.

What these findings *do* indicate is that larger buffers are more likely to predict physical health than smaller buffers, given that these two buffers have equal percentages or densities of green cover. That is, individuals with high densities of green cover in their broader neighborhoods are more likely to have better physical health than individuals with high densities of green cover around their homes but low densities of green cover in their broader neighborhoods.

It should be noted that for smaller and larger buffers to have the same relative amount of green cover requires substantially different actual amounts of green cover. Let us consider a scenario where two buffers both have 50% green cover. The larger buffer has a 1000 m radius and covers a circular area of approximately 3,140,000 m^2^. The smaller buffer has a 250 m radius and covers an area of approximately 200,000 m^2^. Given that both of these buffers have 50% green cover, the 1000 m buffer would include 1,570,000 m^2^ (nearly 400 acres) of greenspace, but the 25 m buffer would only include 100,000 m^2^ (approximately 25 acres) of greenspace. Because the smaller buffer analysis does not describe green cover beyond this relatively small area, it cannot evaluate whether the broader neighborhood includes those additional 1,470,000 m^2^ of green cover represented in the 1000 m buffer analysis. From this example, the power of larger buffers to predict much larger tracts of green space coverage becomes evident. Our findings—that buffers of increasing size better predict physical health than smaller buffers—may, therefore, be the result of larger buffers’ increased power to measure larger tracts of greenspace in a neighborhood. Importantly, though, our finding that buffers of over 2000 m drop in predictive power provides some evidence of a dosage effect of residential greenness. This review suggests that green cover farther away than 2000 m (a 20-min moderately-paced walk) has less impact on physical health than green cover located nearby.

Although this review found that a majority of buffer analyses did *not* report positive findings between greenness and physical health, this finding should not be considered conclusive. Many of the analyses in this review examined health outcomes for which multiple other reviews find consistent ties between greenness and physical health—namely physical activity, birth and developmental outcomes, and cardiovascular outcomes [1,16,23,24,25]. The analyses in this review represent a relatively small sample of articles using a single analytic tool to estimate residential greenness. The low number of positive findings in this review, therefore, is only a slice of the available evidence on the link between nature and physical health.

This review reported several characteristics of buffer analyses that indicate this field of literature is robust and in general is growing stronger. The number of analyses is increasingly nearly every year, and these analyses are being conducted globally—although they are concentrated in Australia, North America, and Western Europe. Very few analyses fail to use objective measures of physical health and controls for income. A considerable number of analyses use substantial sample sizes over 10,000, and the majority of analyses use sizes of at least 2500. These samples represent populations across the lifespan, from infants to elders. The only major limitation we identified is the relatively low number of analyses that consider or control for spatial autocorrelation.

The findings in this review are limited primarily by its scope of studies included. A growing body of GIS buffer analyses considers other health outcomes and may help explain how distance within which greenness is measured influences health. For instance, at least three articles have tied academic achievement to school greenness [26,27,28], and another thirteen articles have tied mental health to residential greenness [18] with buffer analyses. Albeit, the mechanisms by which greenness improves these outcomes may be different than those by which greenness improves physical health. For example, residential greenspace may support physical health primarily by providing opportunities for physical activity [29] or by improving immune functioning [16]. On the other hand, greenspace may reduce anxiety and depression primarily by reducing maladaptive patterns of circular, negative thought called ruminative brooding [30]. Different mechanisms may require different amounts of greenness at different distances from a person’s home. It is feasible that views of trees from windows may be most important for reducing depression—much more so than large green spaces accessible by only a 20-min walk. Even so, it would enhance practitioners’ understanding of the importance of greenspace at different distances from a person’s home address if additional studies with outcomes other than physical health were reviewed in a way similar to that of this study.

We recommend that future researchers using GIS buffers estimate greenspace’s impact on health to try to resolve the unique impact of greenness in nested, rather than overlapping, buffers. All analyses in this review used larger buffer areas that overlapped with smaller ones. As a result, we do not know the unique contribution of farther-away greenspace, that is, the greenspace identified in the *difference* between larger and smaller buffer areas. Analyses that used a nested buffer pattern, wherein a 250 m buffer region is excluded from 500 m buffer analyses, and 250 m and 500 m buffer regions are excluded from 1000 m buffer analyses, would enhance our understanding of at what distance greenness best predicts improvements in physical health.

Another notable recommendation for future research is to use network buffers, which estimate greenness that is physically accessible to people along walking routes. Especially, in the physical activity research domain, the use of network buffers has recently become popular to accurately estimate the impact of greenness on walking [12,13,31]. Other physical health research should apply network buffers in future research to weight the access to reachable greenspaces or parks by walking, which may affect various physical health outcomes.

Also, future research can focus on other GIS methods to define exposure to greenness areas. Recent studies have used GPS units to better understand daily travels of individuals and assess exposures to non-residence environments including workplaces, shopping places, and other areas that play important roles in daily life beyond the neighborhood area immediately surrounding the home [32,33,34,35]. These exposure areas can be estimated using a broad range of different GIS analysis beyond buffers, such as kernel densities and neighborhood delineations [36,37,38]. Which methods provide the most reliable and valid associations between greenspaces and physical health, however, has not been adequately reviewed. Therefore, additional research on physical health findings related to greenness using a variety of GIS methods is warranted.

## 5. Conclusions

This review has demonstrated that greenness measured at larger distances from people’s home environments—specifically buffers between 500 m and 1999 m in size—predicted physical health better than smaller buffers. Because the analyses included in this review used overlapping rather than nested buffers, we were unable to evaluate the impact of farther-away versus closer greenspaces.

## Figures and Tables

**Table 1:** Sample characteristics of analyses (*n* = 260) included in review.

	Number of Analyses	Percent of Analyses
*Size*		
Less than 1000	36	14%
1000 to 2500	41	16%
2501 to 5000	86	33%
5001 to 10,000	12	5%
10,001 to 50,000	26	10%
50,001 to 100,000	9	3%
100,001 to 200,000	4	2%
More than 200,000	46	18%
*Age*		
Infants only	4	2%
Children only	6	2%
Youth only	23	9%
Adults only	79	30%
Elders only	5	2%
Infants and children	3	1%
Children and youth	43	17%
Youth, adults, and elders	50	19%
Adults and elders	47	18%

**Table 2:** Quality of measures in analyses.

	Number of Analyses	Percent of Analyses
*Health data quality*		
Objective	115	44%
Expert or clinical diagnosis	55	21%
Subjective	90	35%
*Greenspace measure*		
Greenness ^a^	124	48%
Green or open space ^b^	110	42%
Park ^b^	23	9%
Tree canopy ^b^	3	1%
*Confounds included*		
Education level	221	85%
Sex	220	85%
Age	209	80%
Smoking behavior	78	30%
Employment status	74	28%
Urbanity	68	26%
BMI	62	24%
Race/ethnicity	60	23%
Marital status	49	19%
Income	44	17%
*Bias Evaluation*		
More likely to be biased	2	1%
Less likely to be biased	114	44%
Least likely to be biased	144	55%

^a^ average value or standard deviation of NDVI; ^b^ percent land cover or number of units (i.e., parks or trees).

**Table 3:** Frequency of analyses finding that greenness improves specific outcomes.

Outcome	Total Number of Analyses	Number with Significant Findings	Percent with Significant Findings
Physical activity (PA)	65	22	34%
Objective PA measures (i.e., accelerometer)	21	10	48%
Subjective PA measures (i.e., self-report exercise frequency)	44	12	27%
Birth and developmental outcomes	41	14	34%
Cardiovascular outcomes	33	6	18%
Obesity	26	13	50%
Atopy	24	1	4%
General health	16	7	44%
Diabetes	12	7	58%
Musculoskeletal complaints	12	4	33%
Cancer	6	4	67%
Mortality	4	4	100%
Upper respiratory tract infection	4	1	25%
Vision	3	3	100%
Acute urinary tract infection	2	1	50%
Infectious disease of intestinal canal	2	2	100%
Migraine	2	1	50%
Respiratory disease	2	0	0%
Vertigo	2	1	50%
Vitality	2	0	0%

## Appendix A

**Table A1:** Search terms used to identify relevant articles for the review.

**Measure of Greenness**
1. Green space
2. Greenness
3. Greenspace *
4. Park *
5. (1 OR 2 OR 3 OR 4) AND
**GIS Analysis**
1. Geographic Information System *
2. GIS
3. Landsat
4. LiDAR
5. NDVI
6. Normalized Difference Vegetation Index
7. Radius
8. (1 OR 2 OR 3 OR 4 OR 5 OR 6 OR 7) AND
**Physical Health Outcome**
1. Allerg *
2. Asthma
3. BMI
4. *Morbidity
5. Mortality
6. Obesity
7. Physical activity
8. Physical health
9. Pregnancy
10. (1 OR 2 OR 3 OR 4 OR 5 OR 6 OR 7 OR 8 OR 9)

* as a wildcard character, the asterisk matches one or more characters.

**Table A2:** Articles included in review.

Authors (Year)	# of Analyses	Sample Characteristics			Greenness Type(s)	Buffer Characteristics			Confound(s)	Health Outcome(s)
		Region(s)	Ages	Size		Type	Size(s)	Center		
Andrusaityte et al. (2016) [39]	3	Lithuania	4–6	1489	Greenness	Radial	100 m 300 m 500 m	Home	Education, smoking, mother’s age at child birth, parental asthma, breastfeeding, antibiotic use, cat, ambient PM2.5, NO_2_	Proportions of children with asthma
Astell-Burt et al. (2014) [40]	2	Australia	45+	203,883	Greenspace	Radial	1 km	Postal code	Age, sex, race, income, education, Body Mass Index (BMI), employment, country of birth, couple status, psychological distress, language other than English, social interactions, neighborhood affluence, geographic remoteness	Walking; Moderate-to-vigorous physical activity (MVPA)
Bijnens et al. (2015) [41]	3	Belgium	Maternal age	211	Greenspace	Radial	3 km 4 km 5 km	Home	Age, sex, education, smoking, birth weight, chronicity, maternal age, neighborhood socio-economic status (SES)	Telomere length
Bodicoat et al. (2014) [42]	6	United Kingdom	20–75	10,476	Greenspace	Radial Network	800 m 3 km 5 km	Postal code	Age, sex, race, BMI, urbanicity, social deprivation, physical activity, fasting glucose, 2 h glucose, total cholesterol	Oral glucose or glycated hemoglobin
Cerin et al. (2017) [13]	4	Belgium, Brazil, Colombia, Czech Republic, Denmark, Hong Kong, Mexico, New Zealand, United Kingdom, U.S.	18–66	6712	Parks	Network	500 m 1 km	Home	Age, sex, marital status, education, employment	MVPA
Chen et al. (2017) [43]	3	United States	9–17	150	Greenness	Radial	250 m	Home	Age, sex, race, income, family relationship, season of visit, asthma severity, atopic status, inhaled corticosteroid use, β agonist use	Asthma control; Asthma functional limitations; T-helper cell expression of glucocorticoid receptors
Dadvand et al. (2012) [4]	9	Spain	16+	2393	Greenness	Radial	100 m 250 m 500 m	Home	Sex, race, education, BMI, smoking, gestational age, maternal age, weight gain during pregnancy, alcohol consumption, parity, season of conception	Birth weight; Birth head circumference; Gestational age
Dadvand et al. (2012) [5]	2	Spain	Maternal age	8246	Greenspace	Radial	100 m	Home	Age, race, education, urbanicity, smoking, employment, gestational age, neighborhood SES, distance of residential place to major roads, maternal booking weight, alcohol consumption, parity, history of obstetrical–gynecological pathologies, diabetes, sex of infant, use of assisted reproductive techniques	Birth weight; Gestational age
Dadvand et al. (2014) [44]	20	Spain	9–12	3178	Greenness	Radial	100 m 250 m 500 m 1 km	Home	Age, sex, education, smoking, having older siblings, type of school, parental history of asthma, sport activity	Asthma; Allergy; Sedentary behavior; Obesity; BMI
Dadvand et al. (2014) [45]	5	United Kingdom	Maternal age	10,780	Greenness	Radial	50 m 100 m 250 m 500 m 1 km	Home	Age, race, education, BMI, smoking, gestational age, neighborhood SES, parity, alcohol consumption, conception year, conception season	Birth weight
Dadvand et al. (2017) [46]	5	Spain	7–10	2727	Greenness	RadialNetwork	50 m 100 m 250 m 500 m	Home/Surrounding school/Commuting school	Age, sex, race, education, smoking, pregnancy period, average screen time per week, Urban Vulnerability Index	Use of spectacles
Demoury et al. (2017) [47]	4	Canada	Under 76	3927	Greenness	Radial	150 m 300 m 500 m 1 km	Home	Age	Prostate cancer risk
Fuertes et al. (2014) [48]	3	Germany	Birth–10	5803	Greenness	Radial	500 m	Home	Age, sex, education, smoking, parental history of atopy, older siblings, cohort	Allergic rhinitis; Eyes and nose symptoms; Aeroallergen sensitization
Fuertes et al. (2016) [49]	2	Sweden, Australia, Netherland, Canada, Germany	6–8, 10–12	13,016	Greenness	Radial	500 m	Home	Age, sex, education, smoking, parental atopy, older siblings, intervention group, cohort, region, birth weight and exposure, to furry pets and mold/dampness in the home	Allergic rhinitis; Aeroallergen sensitization
Ghekiere et al. (2016) [50]	1	Australia	10–12	677	Open space	Radial	800 m	Home	Age, sex, marital status, education, employment	Active transport trips
Gómez et al. (2010) [51]	2	Columbia	60+	1966	Park	Radial	500 m	Postal code	Age, sex, education, having a limitation to engage in physical activity, proximity of a family member, neighborhood SES	Walking
Gong et al. (2014) [52]	2	United Kingdom	66+	1010	Greenness	Radial	400 m	Postal code	Age, marital status, education, urbanicity, car ownership, general health, psychological distress, area deprivation	Participation in physical activity
Grazuleviciene et al. (2015) [53]	15	Lithuania	Maternal age	3292	Greenness	Radial	100 m 300 m 500 m	Home	Age, sex, marital status, education, BMI, smoking, employment, height, diabetes, chronic diseases, parity, gestation duration, previous preterm birth, paternal height, alcohol consumption, blood pressure	Low birth weight; Term low birth weight; Preterm birth; Small for gestational age; Birth weight
James et al. (2016) [54]	4	United States	30–55	108,630	Greenness	Radial	250 m 1250 m	Home	Race, marital status, education, BMI, urbanicity, smoking, employment, pack-years of smoking, neighborhood SES, address change, physical activity, air pollution, social engagement, mental health, region	Nonaccidental mortality
Janssen and Rosu (2015) [55]	2	Canada	11–13	5138	Greenspace	Radial	1 km	Postal code	Sex, race, income, grader, perceived neighborhood characteristics (safety, average income, number of recreational facilities, % of developed parks, playgrounds, total road distances), perceived family wealth, neighborhood average income, survey season	Frequency of physical activity
Kim et al. (2014) [22]	10	United States	9–11	61	Greenness	RadialNetwork	800 m	Home	Age, sex, race, marital status, education, school, grade, guardians, number of cars, country born, environmental perceptions and satisfaction, physical activity	Obesity
Koohsari et al. (2013) [56]	4	Australia	18+	320	Open space	Network	1 km	Home	Age, sex, income, education, employment, neighborhood quality, quality of nearby public open spaces, presence of children less than 12 years in the household, having a dog, residential self-selection	Walking to/within open space
Laurent et al. (2013) [57]	3	United States	Maternal age	81,186	Greenness	Radial	50 m 100 m 150 m	Home	Race, maternal age, poverty, insurance status, parity, pyelonephritis	Preterm birth
Maas et al. (2008) [58]	2	Netherland	12+	4899	Greenspace	Radial	1 km 3 km	Postal code	Age, sex, income, education, urbanicity	Meet the public health recommendations for physical activity
Maas et al. (2009) [21]	44	Netherland	12+	345,143	Greenspace	Radial	1 km 3 km	Postal code	Age, sex, education, urbanicity, employment, healthcare insurance	High blood pressure; Cardiac diseases Coronary heart diseases; Stroke, brain hemorrhage; Neck and back complaints; Severe back complaints; Severe neck and shoulder complaints; Severe elbow, wrist and hand complaints; Osteoarthritis; Arthritis; Upper respiratory tract infection; Bronchi(oli)tis/pneumo nia; Asthma, COPD; Migraine/severe headache; Vertigo; Severe intestinal complaints; Infectious disease of the intestinal canal; MUPS; Chronic eczema; Acute urinary tract infection; Diabetes mellitus; Cancer;
Maas et al. (2009) [59]	6	Netherland	12+	10,089	Greenspace	Radial	1 km 3 km	Postal code	Age, sex, income, education, urbanicity, size of household, social support	General health; Number of health complaints; Propensity for psychiatric morbidity
Markevych et al. (2014) [60]	4	Germany	Birth	3203	Greenness	Radial	100 m 250 m 500 m 800 m	Home	Sex, education, smoking, cohort, year of birth, season of birth, maternal age	Birth weight
Markevych et al. (2016) [61]	12	Germany	10–15	1552	Greenness	Radial	100 m 300 m 500 m	Home	Age, sex, education, BMI, fasting status, physical activity, puberty category, neighborhood SES, study area	Total cholesterol; Low density lipoprotein; Triglyceride; High density lipoprotein
Markevych et al. (2016) [62]	4	Germany	15	1192	Greenness Tree canopy	Radial	500 m	Home	Sex, education, BMI, cohort, accelerometer wear year, season	MVPA
McMorris et al. (2015) [3]	6	Canada	20+	69,910	Greenness	Radial	500 m	Postal code	Age, sex, income, marital status, smoking	Physical activity level; Participants in leisure physical activity; Frequency of physical activity; Physical activity index; Monthly frequency of physical activity; Energy expenditure
Mitchell et al. (2016) [63]	4	Canada	9–14	435	Park	Radial	500 m 800 m	Home	Age, sex, frequent travel mode, siblings, neighborhood SES	MVPA
Paquet et al. (2013) [64]	10	Australia	18+	3751	Open space	Network	1 km	Home	Age, sex, income, education, neighborhood SES	Cardiometabolic risk; Metabolic equivalents (METs)
Pereira et al. (2012) [65]	4	Australia	25+	11,404	Greenness	Network	1.6 km	Home	Age, sex, income, education, BMI, smoking, healthcare card, non-gestational diabetes, hypertension, high cholesterol, daily serves of fruit and vegetables, risky drinking in the last month, air quality	Coronary heart disease
Pereira et al. (2013) [66]	4	Australia	16+	10,208	Greenness	Network	1.6 km	Home	Age, sex, education, smoking, fruit and vegetable intake	Overweight-or-obese; Obese
Picavet et al. (2016) [14]	22	Netherland	20–59	4796	Greenspace	Radial	125 m 1 km	Home	Age, sex, education	MVPA; Physical functioning; Pain; General health; Vitality; BMI; Diabetes; Cardio-vascular diseases; Asthma; Chronic obstructive pulmonary diseases; Systolic blood pressure;
Rundle et al. (2013) [67]	1	United States	Adults	13,102	Park	Radial	805 m	Home	Age, sex, race, education, neighborhood SES	BMI
Sallis et al. (2016) [68]	1	Belgium, Brazil, Colombia, Czech Republic, Denmark, Hong Kong, Mexico, New Zealand, United Kingdom, U.S.	18–66	6822	Park	Network	500 m	Home	Age, sex, marital status, education, employment, accelerometer wear time, neighborhood SES	MVPA
Salvo et al. (2014) [31]	2	Mexico	20–65	677	Park	Network	500 m 1 km	Home	Age, sex, marital status, education, BMI, individual-level SES, vehicle ownership	MVPA
Schipperijn et al. (2013) [69]	6	Denmark	18–80	1305	Greenspace	Radial	100 m 300 m 600 m 1 km 2 km 3 km	Home	Age, sex, education, general health	Participation in physical activity
Scott et al. (2007) [70]	1	United States	12–14	1556	Park	Radial	805 m	Home	Race, Neighborhood SES, number of accessible schools and locked schools, presence of a school within a half-mile, free/reduced price lunch at a school level	METs
Sugiyama et al. (2010) [71]	2	Australia	18+	1366	Open space	Network	1.6 km	Home	Age, sex, presence of children	Recreational walking
Thiering et al. (2016) [72]	2	Germany	15	837	Greenness	Radial	500 m 1 km	Home	Age, income, education, BMI, smoking, physical activity, puberty, cohort, study area	Homeostatic model assessment of insulin resistance
Thornton et al. (2016) [12]	3	United States	66+	726	Parks	Network	1 km	Home	Marital status, BMI, urbanity, smoking behavior, employment status, age, sex, race, income, education, residing in different regions, driver license, health condition, caretaking duty, self-rated mobility impairment, number of people in household, number of vehicles, years at current address, neighborhood indicators (median age, white, median household income)	MVPA; Walking for errands; Walking for leisure
Ulmer et al. (2016) [73]	1	United States	Under 65	4820	Tree canopy	Radial	250 m	Home	Age, sex, race, income, marital status, education, smoking, employment, English language ability, time living at the current address, health insurance, household size, food security status, home ownership, poverty status, survey cycle	General health
Van den Berg et al. (2010) [74]	4	Netherland	19–97	4529	Greenspace	Radial	1 km 3 km	Home	Age, sex, income, education, urbanicity	Number of health complaints; General health
Van Loon et al. (2014) [75]	4	Canada	8–11	366	Park	Network	200 m 400 m 800 m 1.6 km	Home	Age, sex, race	MVPA
Wolch et al. (2011) [20]	1	United States	9–10	3173	Park	Radial	500 m	Home	Sex, race, cohort, AADT density, average urban imperviousness, total length of arterial roads, number of intersections, NDVI, percent below poverty	BMI
